# Fe-Cr-Nb-B ferromagnetic particles with shape anisotropy for cancer cell destruction by magneto-mechanical actuation

**DOI:** 10.1038/s41598-018-30034-3

**Published:** 2018-08-01

**Authors:** H. Chiriac, E. Radu, M. Țibu, G. Stoian, G. Ababei, L. Lăbușcă, D.-D. Herea, N. Lupu

**Affiliations:** 1 National Institute of Research and Developnment for Technical Physics, Iași, Romania; 2 Faculty of Physics, “Alexandru Ioan Cuza” University, Iași, Romania

## Abstract

We introduce a new type of magnetic particles (MPs) prepared by wet milling of superferromagnetic Fe-Cr-Nb-B precursor glassy ribbons for cancer treatment by magneto-mechanical actuation in low magnetic fields (1 ÷ 20 Oe). The rectangular shapes of MPs and the superferromagnetism of the glassy alloys of which are made the MPs induce important magnetic shape anisotropies which, in association with a large saturation magnetization, generate an improved torque in a rotating magnetic field, producing important damages on the cellular viability of MG-63 human osteosarcoma (HOS) cells. The specific parameters such as MPs concentration, frequency and intensity of the applied magnetic field, or the time of exposure have a strong influence on the cancer cells viability. The specific behavior of the Fe-Cr-Nb-B MPs offers them destructive effect even in low magnetic fields such as 10 Oe, and this characteristic allows the use of coils systems which provide large experimental spaces. The novel MPs are used for the magneto-mechanical actuation alone or in association with hyperthermia, but also can be transported to the tumor sites by means of stem cells carriers.

## Introduction

Magnetic particles (MPs) were found useful in different cancer treatment applications, such as magnetic hyperthermia or magnetic controlled delivery and release of antitumoral drugs at the targeted site of a tumor^1–3^. Lately, cancer cell destruction techniques involving the movement of magnetic particles in variable magnetic fields came to the fore^4,5^. Once the destruction effect of moving MPs on tumor cells was demonstrated, the researchers focused their work on finding the most appropriate magnetic materials with larger mechanic torque of MPs under the variable magnetic field action. Thus, magnetite particles^6,7^, magnetic-vortex NiFe microdiscs prepared by magnetron sputtering followed by optical lithography^8,9^, ultrathin perpendicularly magnetized magnetic particles^10,11^, synthetic antiferromagnetic and ferrimagnetic microdiscs^12^ with no magnetic remanence^13^, nanowires with various compositions obtained by electrodeposition in aluminum membranes^14–16^ were studied at large for this purpose. The possibility of using STEM cells as carriers for the MPs to the tumor site, followed by the release of MPs based on magneto-mechanical actuation and destruction of cancer cells was also considered by the scientific world^17^. However, the most suitable type of particles, considering the possibility to scale-up their preparation method, the adequate magnetic field parameters and the optimum geometry for cancer cells destruction applications, were not established so far.

In this work, we aim to test the efficiency of Fe-Cr-Nb-B MPs for magneto-mechanical destruction of cancer cells, MPs that are produced by a controllable process and in a large amount without involving costly technologies. We have in view the superferromagnetic behavior of Fe-Cr-Nb-B glassy alloys^18^ and the specific geometry of the MPs, i.e. the induced shape anisotropy, all leading to the decrease of the magnetic field required to generate important torques of the MPs, and finally the apoptosis of the cancer cells. This systematic study focuses on the effect of the mechanical displacement of our MPs induced by variable magnetic fields on osteosarcoma cells (HOS – human osteosarcoma cells). Linear and rotating variable magnetic fields are used and the influence of the intensity of the field, its frequency and the time of exposure on cellular viability is estimated. They are compared to the effects on a healthy fibroblastic cellular line (NHDF – normal human dermal fibroblasts). Understanding the importance of using MPs, (i) made of superferromagnetic Fe-Cr-Nb-B glassy alloys and (ii) with shape anisotropy, to generate significant torque in low external variable magnetic fields offers a simple and efficient alternative to cure the cancer disease.

## Magnetic Particles and Magnetic Ferrofluid

**Figure 1:**
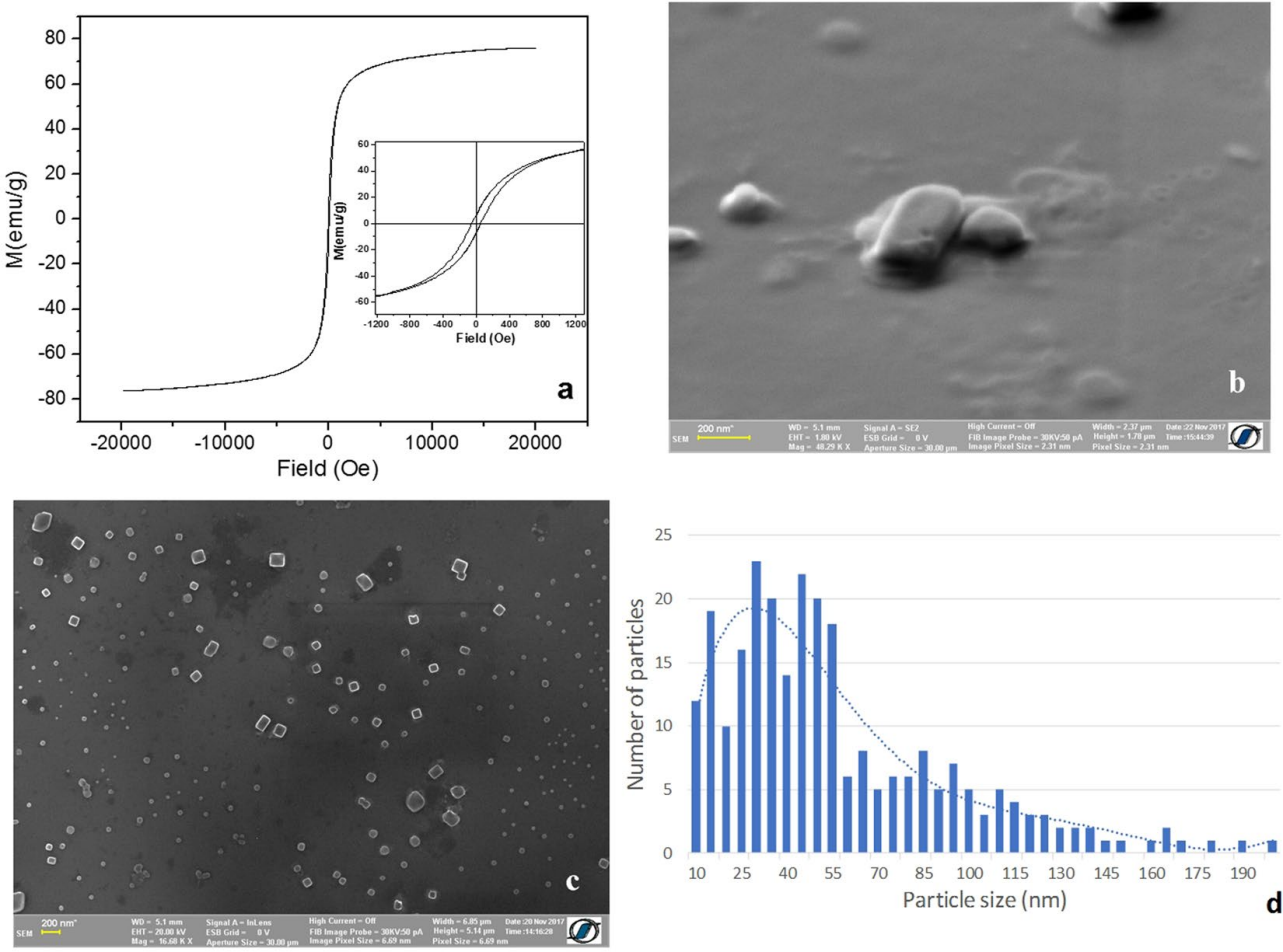
(**a**) M-H loop of Fe_68.2_Cr_11.5_Nb_0.3_B_20_ MPs, showing the non-zero coercivity at room temperature (inset); (**b**–**c**) SEM images of the MPs; (**d**) particles size distribution calculated from the SEM image (**c**).

Fe_68.2_Cr_11.5_Nb_0.3_B_20_ MPs were obtained by high-energy ball milling the superferromagnetic rapidly solidified melt-spun ribbons precursors^18^, and, consequently, the MPs have non-zero coercivity at room temperature (the inset in Fig. 1(a)), contrary to the zero coercivity at room temperature reported in the literature for superparamagnetic Fe-oxide MPs of similar sizes. The MPs exhibit large magnetization, large magnetic susceptibility (Fig. 1(a)) and low Curie temperature of about 47 °C^18,19^. These particles are suitable for self-controlled hyperthermia applications, as described in^19,20^. The preparation process is controllable and allows obtaining of large amounts of particles with reasonable expenses. The size of the particles is ranging between 10 and 200 nm, depending on the milling conditions, and they present non-spherical and irregular shapes (Fig. 1(c,d)). They are rectangular in volume as can be seen in Fig. 1(b,c).

Magnetic particles can be added directly to the cell cultures in powder form or in a suspension as ferrofluid (FF), being manipulated and controlled by an external magnetic field. The advantage of using FF for *in-vitro* applications is that they ensure a good dispersity of MPs in cell cultures and prevent their agglomeration^21^. For our experiments, we have prepared a Fe_68.2_Cr_11.5_Nb_0.3_B_20_ MPs-based FF using calcium gluconate as dispersion media, because of its biocompatibility and good dispersity^22^. To obtain the desired concentration of MPs in cell cultures, the FF was dispersed in cell culture media prior to the experiments. The FF was prepared right before doing the cytotoxicity and cellular viability tests, to avoid the precipitation of large particles, which represents still less than 20% from the total volume of MPs used to prepare the FF (Fig. 1(d)).

### Coil system generating low frequency variable magnetic fields

**Figure 2:**
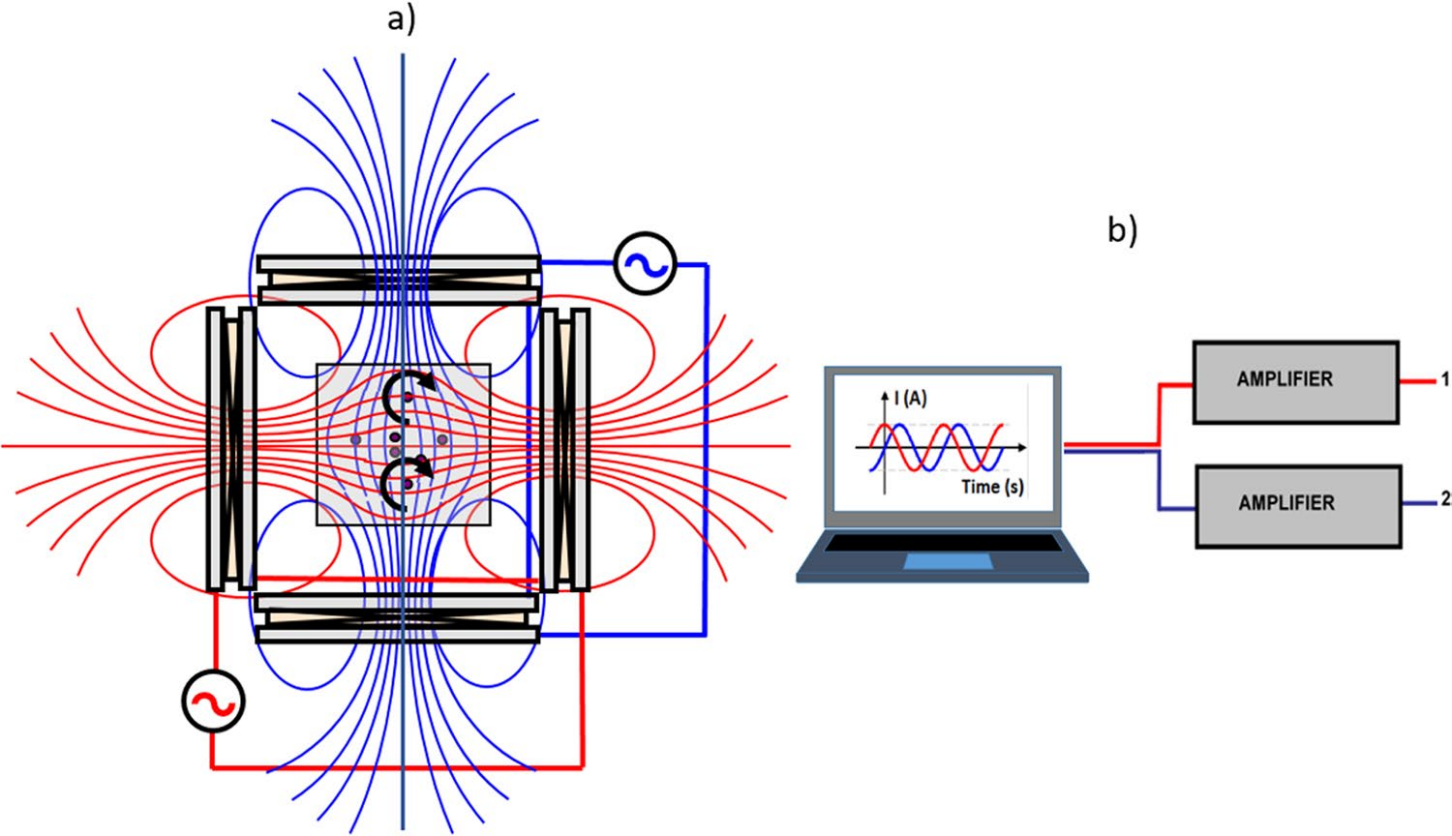
(**a**) The rotating magnetic field produced by an AC current applied in the 4-coil system. (**b**) The set-up for the generation of the AC current to supply the coil system.

To set up the experiments, we designed a system consisting of four coils placed in cross, which can produce either a magnetic field variable in time that generates a high magnetic field gradient or a rotating magnetic field (Fig. 2(a)). The electronics allow to set the magnetic field intensity, its frequency and the time of exposure. In the center of the coil system there is a space of about 20 cm^3^ where the magnetic field is uniform, and the cell culture plates can be placed. A device which allows temperature variation is inserted in order to modify and maintain the desired temperature of the cell plates.

The coil system is fed by two waveforms which are generated using a code realized in LabView and an acquisition board (NI USB-6218) (Fig. 2(b)). The waveforms are phase shifted by 90 degrees in order to fit with the orthogonal arrangement of the coil system, therefore the resultant magnetic field will rotate with the frequency of the waveforms. Depending on the needs, the frequency and amplitude of the waveforms can be adjusted in a wide range from mHz up to several kHz. The generated signals at the analog outputs of the acquisition board are amplified using two identical power amplifiers (based on OPA512). The system generates magnetic fields between 1 Oe and 20 Oe.

The same setup can be used to obtain translation motion for the MPs. For this purpose, only two coils (on the same axis) from the quadrupole are fed by the power amplifier with two independent square waveforms, phase-shifted with 180 degrees and generated by the computer, each coil being connected to the corresponding output.

Such a system of coils can be further modified to allow using MPs magneto-mechanical actuation alone or associated with magnetic hyperthermia and with controlled drug delivery even on tumors situated deep inside the body.

### Cell culture

MG-63 human osteosarcoma (HOS) and Normal Human Dermal Fibroblasts (NHDF) cell lines were purchased from the American Type Culture Collection (Manassas, VA, USA). Both cellular lines were cultured at 37 °C with 5% CO_2_ complete culture media composed by Dulbecco’s Modified Eagle’s Medium (DMEM), 10% Fetal bovine serum (FBS) and 1% Antibiotic-Antimycotic.

**Figure 3:**
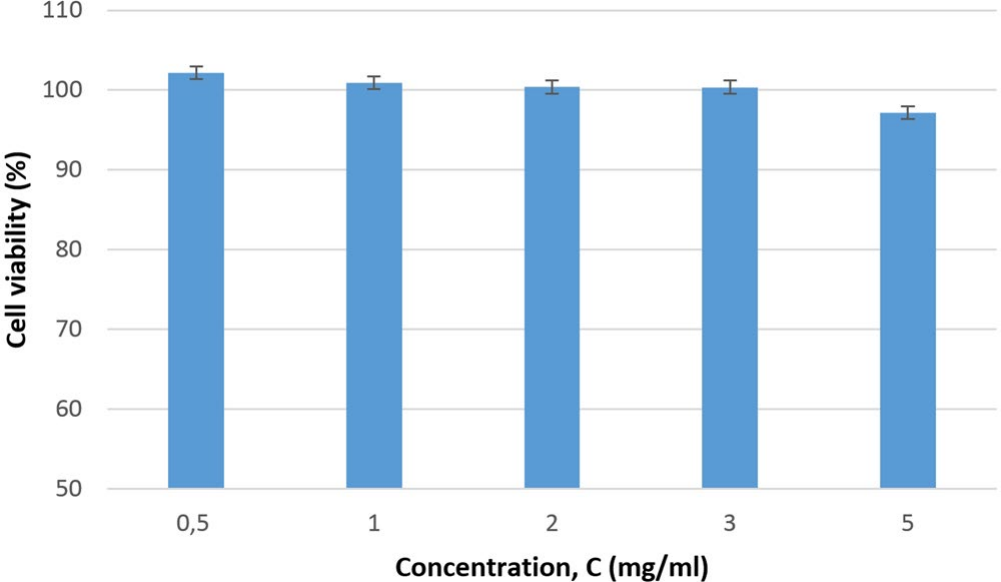
The variation of the cellular viability of osteosarcoma cell controls with concentration of MPs in cell culture media, in the absence of the magnetic field.

Prior to their use in variable magnetic fields, the cytotoxicity of the Fe_68.2_Cr_11.5_Nb_0.3_B_20_ MPs-based FF was evaluated indirectly by measuring the cell proliferation rate for concentrations of MPs in cell culture media ranging between 0.5–5 mg/ml on both cellular types (HOS and NHDF). No cytotoxic effect was observed, even at high concentration rates of 5 mg/ml (Fig. 3).

### Magneto-mechanical actuation

The effect of the movement of MPs induced by a variable magnetic field on HOS was investigated by varying one by one the parameters involved, such as MPs concentration, the frequency and intensity of the magnetic field, and time of exposure. After switching off the magnetic field the cells were maintained for 24 hours at 37 °C with 5% CO_2_, then the MTT assay was performed. First, the MTT assay was performed immediately after the magneto-mechanical exposure, but we have noticed that cellular viability decreases in time and not immediately after the exposure to magnetic field. This is happening, most probably, because the MPs actuation triggers cellular apoptosis, which is not highlighted when MTT assay is performed straight away. The viability of cells is at least 10% lower when the MTT assay is performed after 24 h from exposure, so, in the following experiments the viability assay was performed only 24 h after exposure. Each set of experiments was performed three times and the values have been averaged.

**Figure 4:**
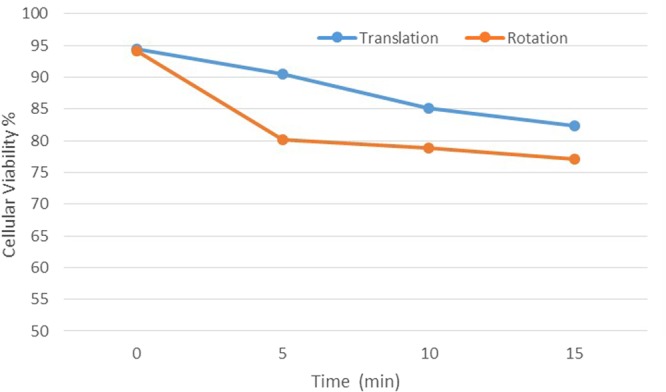
Cellular viability variation of HOS loaded with MPs and exposed to a variable magnetic field which can produce the translation or rotation of the MPs.

We have investigated two different types of actuation of MPs, translation and rotation, depending on the phase shift of the applied waveforms on the quadrupole. As one can see in Fig. 4, the rotating magnetic field configuration has a more efficient impact on cancer cells as compared to the one which determines the translation of the MPs. Such a specific behavior could be explained if we consider that by rotation the magnetic particles are in contact with many cancer cells and destroy them. Consequently, for the following tests we used only the rotating magnetic field.

**Figure 5:**
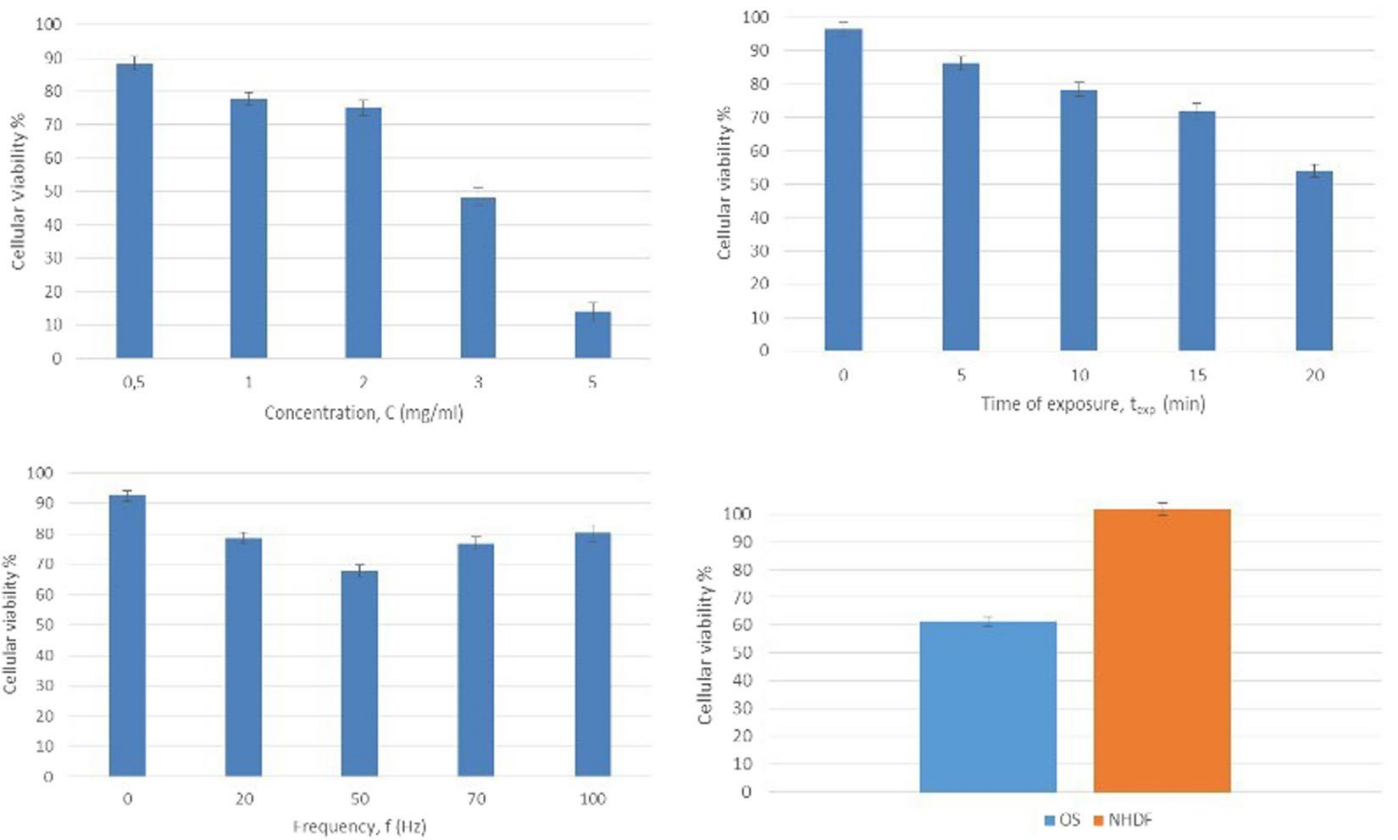
The variation of the cellular viability of osteosarcoma cells with: (**a**) concentration of MPs in cell culture media, after the exposure in variable magnetic field (f = 20 Hz, t_exposure_ = 10 min.); (**b**) time of exposure to variable magnetic field (c = 2 mg/ml, f = 20 Hz); (**c**) frequency of applied magnetic field (c = 2 mg/ml, t_exposure_ = 10 min.). (**d**) Cellular viability of cancerous cells and NHDF after exposure to variable magnetic fields, in the same experimental conditions (c = 2 mg/ml, f = 20 Hz, t_exposure_ = 10 min.).

The effect of MPs concentration, *c*, time of exposure, *t*_*exposure*_, and frequency, *f*, of variable magnetic field on HOS and NHDF cells loaded with MPs have been systematically studied and the results are shown in Fig. 5.

We have tested 5 concentrations: 0.5, 1, 2, 3 and 5 mg of MPs in 1 ml of cell culture for 10 min., at 10 Oe. The various concentrations of MPs in cell culture media have been obtained by dispersing aliquots of ferrofluid in a specific amount of cell culture media, so the final concentration of the suspension is the desired one. The higher the concentration of MPs is the lower the viability of cells after treatment becomes (Fig. 5(a)), reaching 14% cellular viability at a concentration of 5 mg/ml for a time of exposure of 10 min. and a magnetic field of 10 Oe.

The influence of time of exposure on cellular viability has been tested by applying the variable magnetic field for 5, 10, 15 and 20 min. on osteosarcoma cells, at a field intensity of 10 Oe and a frequency of 20 Hz. For each time of exposure, the cells seeded in 24 well plates were tested using a concentration of MPs of 2 mg/ml. The results show that the cellular viability decreases sharply when increasing the time of exposure of the cells to the magnetic field (Fig. 5(b)).

To evaluate the effect of field frequency, we have performed tests at the following frequencies: 20, 50, 70 and 100 Hz, respectively. For each value of the frequency we have exposed HOS cells seeded in 24 well plates and loaded with MPs in 2 mg/ml concentration to a rotating magnetic field with the intensity of 10 Oe. As one can see in Fig. 5(c), the cellular viability decreases with the increase of the frequency, but only to a specific value of the frequency of 50 Hz. Above this value the cellular viability increases with the frequency. This effect is probably caused by the fact that at higher frequencies the MPs cannot follow the rotating magnetic field, due to the viscosity of the medium and the friction forces appearing between MPs and medium, so, the effect of cellular destruction is reduced.

We have also investigated the effect of the magnitude of the applied magnetic field and we have observed that an increase in field intensity leads to a decrease of cellular viability, but only to a certain value of the applied magnetic field. Increasing the field intensity above this value does not produce any significant effect on cells and thus, for the next experiments we have chosen to use the intensity which provoked the most damage on cell culture, namely 10 Oe.

To compare the destruction effects on cancerous cells and normal cells, we have selected the suitable frequency, field intensity, time of exposure and MPs concentration. We have tested three times 24 well plates seeded with 6 wells*2*10^4^/ml of both cell types and 2 mg/ml MPs for 10 min. at 10 Oe. In Fig. 4(d) we can observe the difference between cellular viability of cancerous (HOS) and healthy (NHDF) cells when they have been exposed to the same conditions of variable magnetic field. Both cellular lines were exposed in rotating magnetic field for 10 min. at 20 Hz and 10 Oe, and after 24 hours the MTT assay has been performed. The results show that in the same experimental conditions, cancerous cells viability decreases to 40%, while the viability of healthy cells remains the same (100%), most probably because of the different dynamics of uploaded particles, e.g. the conserved microtubular cytoskeleton system organization in normal cells versus cancer cells. However, supplementary investigations are required to clarify how the MPs subjected to a variable magnetic field are interacting with the healthy cells.

### Visualization of interactions between MPs and cells

**Figure 6:**
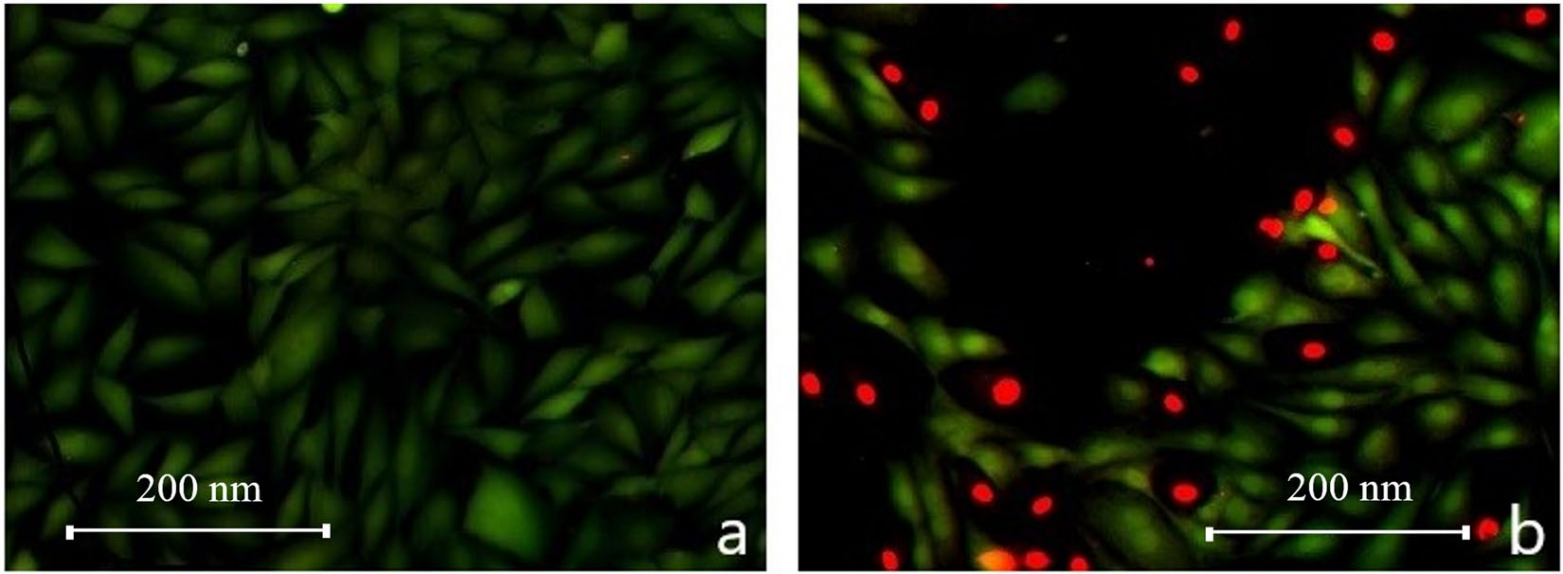
HOS (**a**) before and (**b**) after the magneto-mechanical actuation. Live cells are colored in green and dead cells in red.

We have performed Live/Dead assay after exposure to magneto-mechanical actuation to understand the mechanisms leading to the cellular death. The test measures the cellular viability parameters such as esterase activity and plasma membrane integrity and is used for simultaneous determination of live and dead cells. The assay uses two dyes, Calcein AM, which enzymatically reacts with intracellular esterase activity from live cells, producing an intense green fluorescence, and EthD-1, which enter in cells with damaged membrane, binds with nucleic acids and produces a bright red fluorescence in dead cells^23^. Pictures were obtained separately for live and dead cells and overlaid to see a general aspect of the cellular viability after magneto-mechanical actuation (Fig. 6).

By analyzing the images, one can observe that after the exposure to a rotating magnetic field a significant number of cells have their membrane integrity compromised (red colored – Fig. 6(b)), as compared with Fig. 6(a), where live cells with intact membrane (green cells) are predominant. These results sustain the hypothesis that the magneto-mechanical actuation of MPs leads to the disruption of cellular membrane and, ultimately, to cellular death.

**Figure 7:**
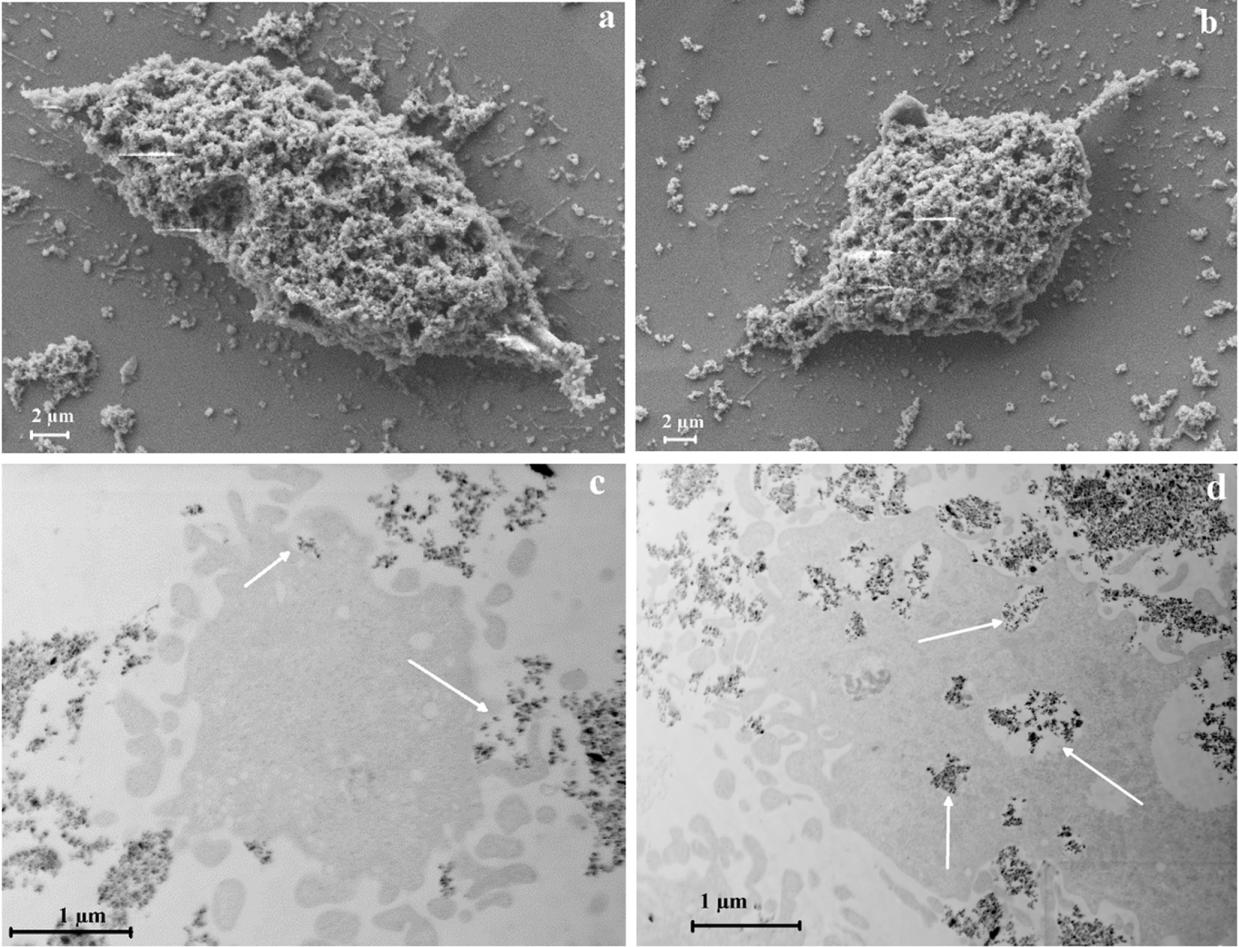
Osteosarcoma cells loaded with MPs: (**a**,**b**) SEM images of HOS covered with MPs; (**c**,**d**) TEM images of HOS loaded with MPs. The particles can be seen both in the extracellular and intracellular medium, being incorporated as single particles or groups of MPs.

To visualize the interactions between MPs and cells and to understand whether the MPs act from inside or outside the cells, we have used scanning (SEM) and transmission (TEM) electron microscopy (Fig. 7). By analyzing SEM images (Fig. 7(a,b)), one could observe that MPs adhere on the surface of cell membrane when incubated together for 24 h.

The interaction between particles and cell membrane seems strong enough, as particles cannot be removed from the cells surface by repeated washing. MPs are incorporated within HOS by means of an active uptake mechanism involving formation of membrane protrusions, indicated by arrows in Fig. 7(c), i.e. by pinocytosis or macropinocytosis in the case of more agglomerated particles. No damage of cellular membranes due to the presence or uptake of particles can be observed at the timepoint of their incorporation (Fig. 7(d)). Further on, the MPs appear to be incorporated within lysosomes and further trafficked inside cell cytoplasm, indicated by arrows in Fig. 7(d), without transpassing cellular organelles membrane.

By applying these techniques on cells loaded with MPs we have observed that MPs not only adhere on the surface of cellular membrane, but they also penetrate inside the cells without damaging the cellular membrane, by being incorporated in lysosomes or by pinocytosis. This leads to the conclusion that the magneto-mechanical actuation on cellular viability is the result of the interaction at membrane level and of the MPs contained in cells cytoplasm.

Many hypotheses were generated up to now to explain how the mechanical forces produced by magnetic microdisks or nanoparticles in low frequency magnetic fields lead to loss of cells membrane integrity. In a previous study, it was demonstrated that calcium level in cells increases after the magneto-mechanical actuation, leading to the hypothesis that mechanical stimuli produced by the microdisks are converted at cellular level into chemical ionic signals that trigger apoptosis and cells death^4^. Another study sustains that the magneto-mechanical stimulation of cellular membrane through MPs results in the destruction of cellular membranes and activation of mechano-sensitive channels of the membrane surface^9^. *Zhang et al*. also showed that the nanoparticles internalized into intracellular medium by lysosomes rotate under a dynamic magnetic field and induce shear forces that tear the lysosomal membrane, followed by lysosomal content leakage into cytoplasm, decrease of cellular pH and, finally, apoptosis^24^.

We have demonstrated that magneto-mechanical actuation of MPs leads to cellular destruction. The effect depends on the shape and nature of the used MPs, their concentration in cell culture medium, the type of particle’s actuation and external magnetic field characteristics, i.e. intensity, frequency and time of exposure. The induced actuation into MPs by the applied MF generates forces strong enough to disrupt cellular or lysosomal membrane and to induce ionic balance disturbance which eventually leads to apoptosis and magneto-mechanical cell destruction.

### Magneto-mechanical actuation and hyperthermia

**Figure 8:**
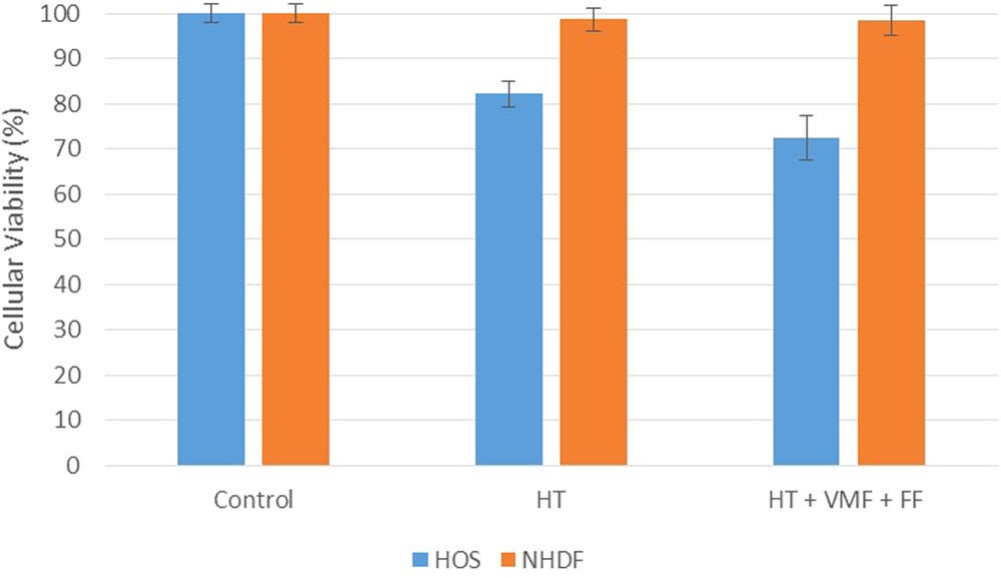
HOS and NHDF viability after applying hyperthermia and magneto-mechanical actuation. HT - conventional hyperthermia, FF - ferrofluid, VMF – variable magnetic field. Identical experimental conditions were maintained for all the samples.

First, we have checked if a simultaneous magneto-mechanical actuation of MPs in variable magnetic fields and hyperthermic treatment is leading to a decrease of the cellular viability as compared with hyperthermic regime only (Fig. 8). The heating effect have been induced by a laboratory-made incubation system, and the temperature of the cell culture well have been raised from 37 °C to about 46 °C. The magneto-mechanical exposure has been applied for 3 min. from the moment the temperature reached 45 °C in the well. The results show that the viability of osteosarcoma cells is reduced to ~80% for a conventional hyperthermic regime and decreases to ~70% when hyperthermia and the variable magnetic field are applied simultaneously. It is worth noting that NHDF are not affected by hyperthermia or variable magnetic field combined with hyperthermia.

### Mesenchymal stem cells loaded with magnetic particles

Recently, stem cells have been proposed as suitable carriers for tumor-targeting therapeutics^25^. In our previous work^26,27^, we have investigated the interaction of Fe-Cr-Nb-B MPs with human adipose-derived mesenchymal stem cells (MSCs) and human bone marrow-derived MSCs. It was observed that Fe-Cr-Nb-B MPs have been internalized by both types of cells and did not induce *in vitro* cytotoxicity, indicating a good biocompatibility. Moreover, MPs internalization did not interfere with proliferative and differentiation potential (adipogenesis and osteogenesis) demonstrating an unaltered phenotype in the adult human MSCs investigated. In a more recent unpublished work, we have observed that human adipose-derived MSCs have been able to migrate *in vitro* towards tumor-like structures after internalizing large amounts of MPs functionalized with an antitumor drug. In this work, we loaded MSCs with MPs and exposed them to a rotating magnetic field with a frequency of 20 Hz for 10 min. We observed that after the exposure the viability of MSCs decreases and the microscopic inspection of the cells shows the shrinkage of the cells and cellular debris containing MPs in the cell culture. In this context, Fe-Cr-Nb-B MPs functionalized with antitumor drug and combined with MSCs can be used as carriers towards tumor sites, offering novel modalities for the treatment of multi-faced antitumor grounded on magneto-mechanic actuation effects conjugated with chemotherapy and, finally, with magnetic hyperthermia.

## Conclusions

We have studied the magneto-mechanical actuation the Fe-Cr-Nb-B MPs undergo when exposed to a rotating magnetic field and the damages produced on cellular viability of HOS cells. We have emphasized the influence of specific parameters such as MPs concentration, frequency and intensity of the applied magnetic field, and the time of exposure by the instrumentality of a new type of MPs prepared by wet milling of Fe-Cr-Nb-B precursor superferromagnetic glassy ribbons. The rotation of MPs induced by exposure of HOS cells loaded with particles has a significant decreasing effect on cellular viability. The rectangular shapes of MPs and the superferromagnetism of the glassy alloys of which are made the MPs induce important magnetic shape anisotropies which, in association with a large saturation magnetization, generate an improved torque. The specific behavior of the Fe-Cr-Nb-B MPs offers them destructive effect even in low magnetic fields such as 10 Oe, and this characteristic allows them to be used in coils systems which provide large experimental spaces. The novel MPs could be used for the magneto-mechanical actuation alone or in association with hyperthermia, but also can be transported to the tumor sites by means of stem cells carriers.

## Methods

### Preparation of Fe-Cr-Nb-B magnetic particles

The preparation and characterization of Fe_68.2_Cr_11.5_Nb_0.3_B_20_ superferromagnetic glassy alloys is described in our papers^18–20^. MPs are obtained by high energy wet ball milling in planetary ball mill operating at 550 rpm, from melt-spun ribbons precursors 20–25 μm thick and 1–2 mm width prepared by rapid quenching from the melt, using oleic acid as surfactant^19^. During the milling process, the pieces of ribbon are reduced depending on milling conditions, but the initial rectangular shape is preserved. By controlling the process, we can downsize the particles to 10–200 nm.

### Ferrofluid preparation

To obtain the ferrofluid, 150 mg of Fe-Cr-Nb-B MPs have been washed 3 times with NaOH 5% to remove the Sodium Oleate excess remained from the milling process, and then with deionized water until it reached pH = 7. MPs have been separated from the solution with a magnet, the water was removed and replaced with 1,5 ml calcium gluconate solution, 80 mg/ml. The temperature of the solution has been raised to 80 °C and the mixture was ultrasonicated for 30 min. by using an ultrasonic probe. The resulting ferrofluid, with a concentration of 50 mg/ml was fractionated in several aliquots and sterilized at 121 °C for 30 min., by using an autoclave.

### Cell seeding and MTT assay

HOS cells have been seeded in 24 or 96 well plates and left to adhere overnight. The culture media was then replaced in some of the wells with fresh media containing ferrofluid dispersed in the desired concentration and exposed in magnetic field after 30 min. The plates were divided in 3 categories: (i) cells with no MPs exposed in magnetic field, (ii) cells with MPs which were not exposed to magnetic field, and (iii) cells with/without MPs exposed to an alternative magnetic field. 24 h after exposure, the MTT (3-(4,5-dimethylthiazolyl-2)-2,5-dyphenyltetrazolium bromide) assay was performed in order to evaluate the reduction of cellular viability. MTT assay is based on the reduction of tetrazolium salts by metabolically active cells through dehydrogenase enzymes and the intracellular purple formazan resulted can be solubilized and quantified by spectrophotometric methods^28^.

The cellular viability of the sample (%) was calculated using the following equation:

$${\rm{CV}}\,( \% )=100\times \frac{{{\rm{OD}}}_{{\rm{MPs}}}-{{\rm{OD}}}_{{\rm{Blank}}}}{{{\rm{OD}}}_{{\rm{Control}}}-{{\rm{OD}}}_{{\rm{Blank}}}}$$

where CV (%) represents cellular viability and OD represents the optical density of the wells containing (a) cells with magnetic particles dispersed in culture medium (OD_MPs_), (b) cells only (OD_Control_) and (c) culture medium without cells (OD_Blank_). The absorbance of the samples was measured at 570 nm by using Multi-Mode Microplate Reader Synergy HTX.

### Live/Dead assay

For this assay, after applying the magneto-mechanical actuation, the cells have been washed with PBS, incubated for 30 min. at room temperature with diluted LIVE/DEAD assay reagents and visualized under microscope. The fluorescence emission for calcein AM was visualized using a filter at 530.12.5 nm and for EthD-1 at 645 + 20 nm, then the images have been overlaid to observe an overview of the magneto-mechanic actuation on the cell culture.

### Electron microscopy for MPs loaded HOS samples

For SEM imaging we have prepared samples by a protocol described in^29^. Briefly, HOS cells have been grown on sterilized silicon chips placed in wells of a 24-wells plate until 80% confluence and incubated with MPs for 24 h. Then the surface of the chip has been washed with PBS in order to remove the non-attached MPs and the cells were fixed with 2,5% glutaraldehyde in PBS for 60 min., rinsed and post-fixed with 1% osmium tetraoxide in PBS for another 60 min., then dehydrated with a graded ethanol series of 25, 50, 75, 95 and 100%. For full dehydration of the sample, hexamethyldisilazane was used as an alternative for critical point drying.

Preparation of samples for TEM has been performed by using a protocol described by *Schrand et al*.^30^. For this, HOS cells were grown in 25 ml flask (Corning) to 80% confluence and incubated with 100 µg/ml MPs for 24 h. After the incubation time, the supernatant from the flasks has been removed and the cells washed three times with PBS to remove the excess particles, then tripsinized and centrifuged for 5 min. at 300 g to form a pellet on the bottom of a Corning 15 ml centrifuge tube. The pellet was treated with a mixture of fresh 2,5% glutaraldehyde/paraformaldehyde in PBS and left to penetrate the sample for 2 h. at room temperature. After the fixation was complete, the sample was rinsed with PBS and treated with 1% osmium tetra oxide in PBS for 1 h, then the fixative was removed by repeated washing of the probe with PBS and water. The dehydration of the probe has been performed by using a graded series of ethanol concentrations (50, 70, 90 and 100%), followed by embedding the sample in Epoxy Resin. After the cells have been embedded in resin, they have been cut with an ultramicrotome and visualized with TEM.
